# The Impact of Artificial Intelligence on the Preference of Radiology as a Future Specialty Among Medical Students at Jazan University, Saudi Arabia: A Cross-Sectional Study

**DOI:** 10.7759/cureus.41840

**Published:** 2023-07-13

**Authors:** Khalid M Hakami, Mohammed Alameer, Essa Jaawna, Abdulrahman Sudi, Bahiyyah Bahkali, Amnah Mohammed, Abdulaziz Hakami, Mohamed Salih Mahfouz, Abdulaziz H Alhazmi, Turki M Dhayihi

**Affiliations:** 1 Faculty of Medicine, Jazan University, Jazan, SAU; 2 Faculty of Medicine, Jazan University, jazan, SAU

**Keywords:** jazan university, intervention, career choice, radiology, medical education, artificial intelligence

## Abstract

Background

The use of artificial intelligence (AI) in healthcare continues to spark interest and has been the subject of extensive discussion in recent years as well as its potential effects on future medical specialties, including radiology. In this study, we aimed to study the impact of AI on the preference of medical students at Jazan University in choosing radiology as a future specialty.

Methodology

An observational cross-sectional study was conducted using a pre-tested self-administered online questionnaire among medical students at Jazan University. Data were cleaned, coded, entered, and analyzed using SPSS (SPSS Inc., USA) version 25. Statistical significance was defined as a P-value of less than 0.05. We examined the respondents' preference for radiology rankings with the presence and absence of AI. Radiology's ranking as a preferred specialty with or without AI integration was statistically analyzed for associations with baseline characteristics, personal opinions, and previous exposures among those who had radiology as one of their top three options.

Results

Approximately 27.4% of males and 28.3% of females ranked radiology among their top three preferred choices. Almost 65.2% were exposed to radiology topics through pre-clinical lectures. The main sources of information about AI for the studied group were medical students (41%) and the Internet (27.5%). The preference of students for radiology was significantly affected when it is assessed by AI (P < 0.05). Around (16.1%) of those who chose radiology as one of their top three choices strongly agree that AI will decrease the job opportunities for radiologists. Logistic regression analysis showed that being a female is significantly associated with an increased chance to replace radiology with other specialty when it is integrated with AI (Crude odds ratio (COR) = 1.91).

Conclusion

Our results demonstrated that the students’ choices were significantly affected by the presence of AI. Thereover, to raise medical students' knowledge and awareness of the potential positive effects of AI, it is necessary to organize an educational campaign, webinars, and conferences.

## Introduction

In recent times, there has been comprehensive discussion about improving medical services when artificial intelligence (AI) technology is used [1], and the experience of using AI in healthcare continues to be improved. The experience extended to many specialties, such as radiology [2]. Machine learning (ML) is a practical field of AI to develop software that can automatically learn from prior data, build up knowledge from experience, and gradationally enhance its learning behavior to make predictions based on new data [3].

AI is likely to play a prominent part in medicine and healthcare. It was found that the healthcare market for AI is increasing at a rate of 40% and is expected to reach $6.6 billion by 2021 [4]. The integration of AI and ML has become an area of interest for many specialties, and in radiology, about 800 publications discussed this topic in 2017 [5]. AI is helpful in numerous ways, and it could support multiple weaknesses in current medical practice and reduce the repetitive work of healthcare providers [6]. AI may also decrease the number of errors in clinical practice and improve clinical judgments among medical professionals. New patterns could be discovered along with new biomarkers for diagnosis and treatment [7].

Deep learning (DL) is another part of ML models that, based on deep convolutional neural networks, has shown high potential in medical image processing [8,9]. DL is very popular today because they obtain tremendous results even at the human-level performance [10]. The best practice examples include categorizing chest radiographs based on abnormality for triage [11] or pathological processes [12,13]. Others include classifying skin cancer with competence comparable to human efforts by dermatologists and promising results in identifying diabetic retinopathy and associated eye diseases [14,15]. It was suggested that radiology is one of the medical sectors that would be most affected by AI, which substitutes specialists in radiology [7]. Lately, experts in AI have warned that radiologists may soon be out of a job, or supervising DL [16].

A Canadian national study revealed that approximately half of the aspiring radiologists expressed concerns about AI, about one-third of medical students thought that AI would replace radiologists, and one-sixth of medical students denied their choice for radiology because of AI [1].

Although AI algorithms have been shown to outperform human radiologists in certain specific tasks, experts in the field agree that a radiologist's complex work requires general intelligence and, therefore, cannot be worked out by narrow AI solutions only [1]. In the foreseeable future, radiology may move to a model where radiologists are boosted with AI. Still, full, integration of AI solutions into routine radiology workflow could be delayed by obstacles in IT infrastructure, data curation, and regulatory approval [1,17].

Medical students and aspiring radiologists may tend to develop an unfavorable view of the future of radiology as a specialty to consider. Thus, the present study aims to assess medical students' awareness and perceptions regarding the utilization of AI in radiology, as well as explore the factors influencing their career intentions toward radiology as a future specialty, both in the presence and absence of AI.

## Materials and methods

Study design, setting, and population

An observational cross-sectional study was conducted using a self-administered anonymous electronic survey aimed to assess the impact of AI on the preference for radiology as a future specialty by medical students at Jazan University, Jazan province is located in the southwest corner of Saudi Arabia and populated with about two million habitants.

Inclusion and exclusion criteria

Our study targeted the medical students at Jazan University with the following inclusion criteria; being a medical student from the second year to the sixth year of college, and internship. Those in the preparatory year or who refused to participate were excluded from the study.

Sample size and technique

The sample size for this study was determined to be 368 participants using http://www.raosoft.com/. Based on Jazan University's annual report states that there are 8,424 students enrolled in health colleges including the College of Medicine and Surgery. The study uses the parameters of the prevalence of 50%, 95% confidence interval (CI), and error not exceeding 5% to produce a maximum sample size. In addition, a 25% non-response rate was taken into account for this study. The sample for this study was selected using a convenience sampling method.

Study instrument and data collection

Using a self-administered anonymous electronic survey prepared in English obtained from a previously published study after taking their consent to use it. The survey was sent through official email to the included students [18]. The survey took about three to four minutes to be filled out, it was distributed by the study’s authors as well as data collectors from each year in the college through different social media such as Telegram, WhatsApp, and Twitter. The questionnaire is divided into three sections.

The first section includes data about baseline characteristics (age, gender, year in medical school, radiology ranking as a preferred specialty compared to other specialties, whether integrated with and without AI influence, and if they are interested in diagnostic or interventional radiology. The second section sought medical students’ level of understanding of AI and its impact on their specialty choices, where they were exposed to radiology, as well as their source of information about AI. The third section evaluated the benefits and uses of AI in radiology from medical students' perspective and how that could affect their specialty choices.

Ethical consideration

This study was approved by the ethical committee of Jazan University, before starting the work with reference no. (REC-43/10/240). The survey does not acquire private information from the participants and the data were collected considering maximum privacy, safety, and confidentiality. The participation was entirely voluntary, and refusal to participate at any point by closing this survey involved no penalty or loss of benefits. Consent was taken before beginning the survey if students voluntarily participated.

Pilot study

After ethical approval was obtained, a pilot study was carried out including 10% of the sample size to test the validity and accuracy of the questionnaire applied to respondents. Some improvements and rearrangements of some questions were made depending on the pilot study results. The final data analysis did not involve the findings of the pilot study.

Statistical analysis

Data were analyzed using SPSS version 25 (IBM Corp., Armonk, NY). Descriptive and inferential statistical techniques were used for data analysis. For descriptive categorical variables, proportions were computed. Likert scale questions were subject to description using the frequency distribution. Chi-squared test was used to test for the statistical significance of associations between some categorical variables. Non-parametric statistical tests were used because of the non-normality distribution of students’ responses based on the Kolmogorov-Smirnov test. In addition, the Mann-Whitney U and Wilcoxon matched-pairs signed rank tests were used to compare radiology ranking by the medical students. Univariate logistic regression was used to assess the independent predictors of change in radiology ranking due to AI using some associated factors. Crude odds ratios (CORs) and their 95% CI were computed. All significance tests were two-sided with a p-value less than 0.05 as an indicator of statistical significance.

## Results

A total of 491 participants responded to the questionnaire and were included in this study. Out of 491 participants, 270 (55.2%) were males. The majority of participants 203 (41%) belong to the age group 20-22 years. Second-year students represented the largest group 132 (27%), followed by fifth-year students 96 (20%). One hundred thirty-seven students ranked radiology as one of their top three specialty choices. One hundred seventy eight (36%) of study participants had an interest in diagnostic radiology (Table 1).

**Table 1:** Background characteristics and radiology top three choices of the survey respondents (n=491).

Characteristic		All participants		Radiology top 3 choices		P-value
		N	%	N	%	
Gender	Male	270	55.2	74	27.4	0.765
	Female	219	44.8	62	28.3	
Age groups (years)	18-20	120	24.4	41	34.2	0.437
	20-22	203	41.3	53	26.1	
	22-24	136	27.7	33	24.3	
	25-26	20	4.1	6	30.0	
	Older than 26	12	2.4	4	33.3	
Academic year	2nd	132	26.9	52	39.4	0.001
	3rd	72	14.7	17	23.6	
	4th	81	16.5	23	28.4	
	5th	96	19.6	21	21.9	
	6th	42	8.6	15	35.7	
	Internship	68	13.8	9	13.2	
Radiology as a preferred specialty	Diagnostic	178	36.3	75	42.1	<0.001
	Interventional	121	24.6	40	33.1	
	Unsure	58	11.8	17	29.3	
	Not considering radiology	134	27.3	5	3.7	

Almost 320 (65.2%) were exposed to radiology topics through pre-clinical lectures, 118 (24%) through research, and 109 (22.2%) through medical school interest. Students who chose radiology as one of their top three choices had an exposure rate to radiology through required clerkship rotation, elective rotation, medical school interest, shadowing, research, and conferences than other students, the difference between the two groups was statistically significant (P < 0.05). Regarding exposure to AI, other medical students were the most popular source among students (41.5%) followed by media/internet (27.5%). Participants who had radiology as one of their top three choices were more exposed to AI than other students through radiology and non-radiology attendings/residents, academic journals, medical students, family, and research experience, and these differences were significant except for medical students, media/internet, and research experience as shown in Table 2.

**Table 2:** Participants' exposure to radiology and artificial intelligence (n=491).

Factors	Proportion with Yes response						P-value
	All Students		Students who put Radiology as Top 3 Choices		Other students who did not prefer radiology		
	N	%	N	%	N	%	
Exposure to radiology							
Preclinical lectures.	320	65.2%	80	58.4%	240	67.8%	0.050
Required clerkship rotation.	96	19.6%	40	29.2%	56	15.8%	0.001
Elective rotation	88	17.9%	39	28.5%	49	13.8%	<0.001
Medical school interest	109	22.2%	49	35.8%	60	16.9%	<0.001
Shadowing.	60	12.2%	34	24.8%	26	7.3%	<0.001
Research.	118	24.0%	49	35.8%	69	19.5%	<0.001
Conferences.	41	8.4%	21	15.3%	20	5.6%	0.001
Exposure to artificial intelligence							
Radiology attendings/residents.	119	24.2%	42	30.7%	77	21.8%	0.039
Non-radiology attendings/residents.	69	14.1%	36	26.3%	33	9.3%	<0.001
Academic journals.	100	20.4%	43	31.4%	57	16.1%	<0.001
Medical students	204	41.5%	65	47.4%	139	39.3%	0.099
Family.	94	19.1%	39	28.5%	55	15.5%	0.001
Media/Internet.	135	27.5%	37	27.0%	98	27.7%	0.880
Research experience.	68	13.8%	25	18.2%	43	12.1%	0.079

According to Table 3, 8.8% of students who chose radiology as one of the top three choices strongly agree that they have a good understanding of the field of radiology, compared to only 2.3% of the general students with a significant difference between the two groups (P < 0.05). Further 10.9% of the students who chose radiology as one of the top three choices strongly agree that they have a good understanding of how AI will be used in radiology, while 4% of other students strongly agreed (P < 0.05). 16.1% students who chose radiology as one of the top three choices strongly agree that there will be a decrease in job opportunities for radiologists due to replacement by AI in their lifetime. 22.6% of students who chose radiology as one of the top three choices strongly agree that AI will increase the efficiency of practicing radiologists, compared to 24.6% of the general student.

**Table 3:** Medical students who put radiology on top three choices compared to other, subjective opinions on radiology and AI based on a 7-point Likert scale.

Statement	Radiology Ranking	Strongly disagree	Disagree	Somewhat disagree	Neither agree or disagree	Somewhat agree	Agree	Strongly agree	P-value
I have a good understanding of the field of radiology.	Top 3 Choices	5.1%	9.5%	17.5%	27.7%	22.6%	8.8%	8.8%	<0.001
	General Students	10.7%	18.1%	21.2%	26.3%	15.5%	5.9%	2.3%	
I have a good understanding of how artificial intelligence will be used in radiology.	Top 3 Choices	7.3%	7.3%	15.3%	32.1%	17.5%	9.5%	10.9%	<0.001
	General Students	15.3%	18.6%	21.5%	20.9%	13.6%	6.2%	4.0%	
I am concerned about considering radiology as a specialty due to artificial intelligence.	Top 3 Choices	5.8%	10.9%	11.7%	28.5%	15.3%	16.8%	10.9%	<0.001
	General Students	19.2%	15.8%	13.8%	26.8%	12.7%	8.2%	3.4%	
There will be a decrease in job opportunities for radiologists due to replacement by artificial intelligence in my lifetime.	Top 3 Choices	6.6%	9.5%	14.6%	29.9%	14.6%	8.8%	16.1%	0.845
	General Students	6.8%	11.9%	13.8%	23.4%	20.1%	14.1%	9.9%	
Artificial intelligence will increase the efficiency of practicing radiologists.	Top 3 Choices	2.9%	8.8%	5.1%	28.5%	16.8%	15.3%	22.6%	0.845
	General Students	2.8%	5.9%	11.6%	20.3%	17.2%	17.5%	24.6%	

**Figure 1:**
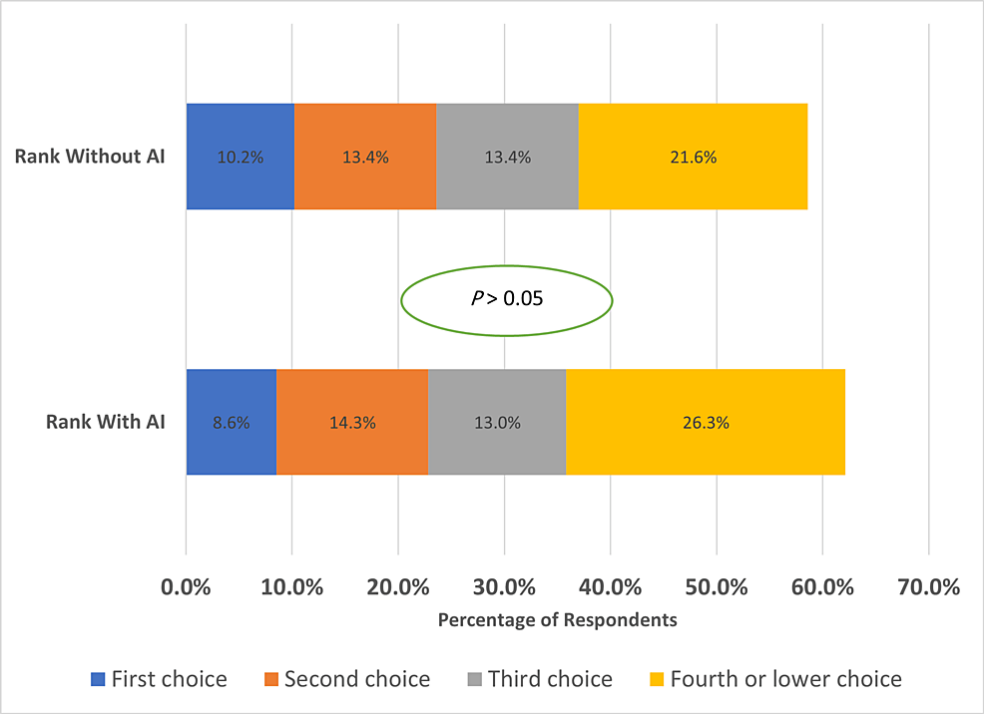
Radiology ranking by medical students with and without AI.

Figure 1 shows radiology ranking as a preferred choice by medical students with and without AI. Participants ranked radiology without AI as a first-choice accounting for 10.2%, while 8.6% ranked it as a first choice with AI. Further, 13.4% ranked radiology with AI as a second choice, while 14.3% ranked it with AI as a second choice. The difference in ranking radiology with and without AI was statistically significant (P < 0.05).

**Figure 2:**
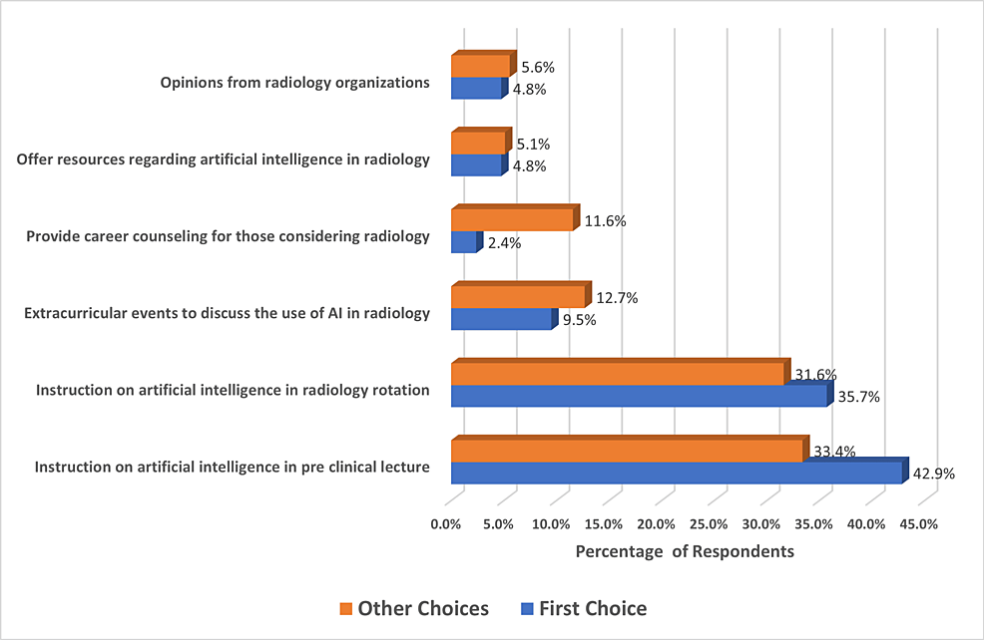
Participants' views regarding AI education in radiology with respect to specialty choice.

Figure 2 demonstrated that 42.9% of students who chose radiology as a first choice think that information about AI in pre-clinical lectures will be the most beneficial way for medical students to learn about the use of AI in radiology, while 33.4% of other students agreed on that. 35.7% of students who chose radiology as a first choice think that information on AI in radiology rotation will be the most beneficial, and 9.5% think that extracurricular events to discuss the use of AI in radiology, 2.4% think of providing career counseling for those considering radiology, 4.8% agreed on offering resources regarding AI in radiology, and 4.8% think that opinions from radiology organization will be the most beneficial way.

Logistic regression analysis showed that being a female is significantly associated with an increased risk of changing radiology as a preferred specialty due to AI by 1.9 times (COR= 1.91). No other significant predictors were noticed (Table 4).

**Table 4:** Logistic regression analysis of independent predictors of changing radiology as a preferred specialty due to artificial intelligence. COR = Crude odds ratio, OR = estimated odds ratio, 95% CI = a 95% confidence interval.

Variables	P-value	COR	95% CI for (OR)	
			Lower	Upper
Gender				
Male(ref)				
Female	0.040	1.91	1.03	3.53
Academic year				
Pre-Clinical	0.219	2.61	0.57	12.08
Clinical	0.462	1.79	0.38	8.43
Internship(ref)				
Exposure to radiology				
Shadowing.	0.295	1.47	0.71	3.04
Research.	0.201	1.51	0.80	2.83
Exposure to artificial intelligence				
Radiology attendings/residents. .	0.060	0.47	0.22	1.03
Non-radiology attendings/residents.	0.057	1.96	0.98	3.91
Family	0.231	1.50	0.77	2.92
Research	0.779	1.12	0.50	2.55

**Figure 3:**
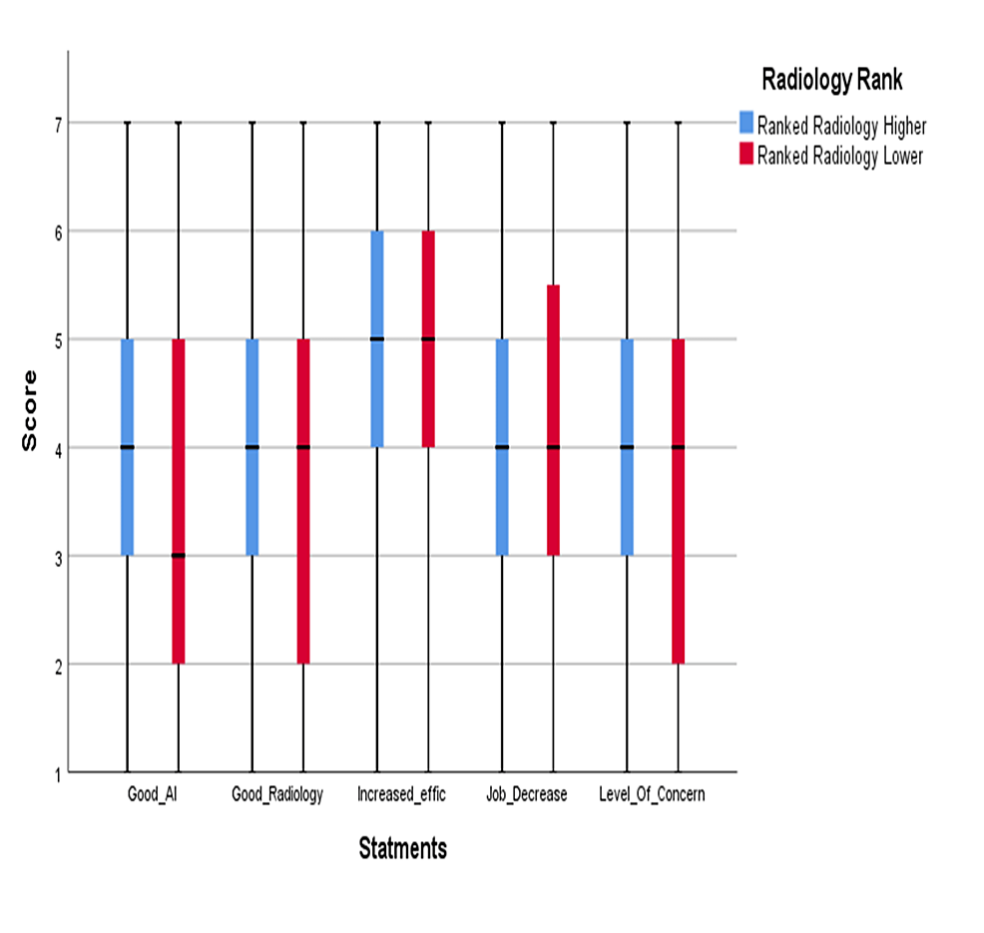
Medical students' subjective opinions on radiology and AI based on a 7-point Likert scale according to radiology ranking.

Significant difference between the two groups in good understanding of AI and concerned about choosing radiology due to AI (P < 0.05) for both based on Mann-Whitney U test as shown in Figure 3.

## Discussion

Rapid advances in the field of radiology would certainly revolutionize the practice of radiologists, as routine tasks to be performed faster and with more efficiency with the aid of AI. However, some radiologists’ tasks can be complicated for AI, such as resolving complicated clinical cases.

Medical students need to understand the impact of AI and its implications in the field of radiology to make a rational decision about radiology as a future specialty. Thus, this study aimed to assess the impact of AI on medical students' preference of choosing radiology as a future specialty.

In this study, 491 participants were included, and 137 students had chosen radiology as one of their top three specialty choices. About 36.3% (178) of study participants had an interest in diagnostic radiology. Our results showed that 320 (65.2%) of the study participants were exposed to radiology through pre-clinical lectures, 118 (24%) through research, and 109 (22.2%) through medical school interest. This is justified as radiology is already embedded in the education curriculum of most medical schools. Regarding exposure to AI, other medical students were the most popular source among students (41.5%) followed by media/internet (27.5%), owing to the fact the study participants belong to the modern generation where technology and electronic devices thrive.

Our results revealed that 8.8% of students who chose radiology as one of the top three choices confirm that they have a good understanding of the field of radiology. In addition, 10.9% of students who chose radiology as one of the top three choices strongly agree that they have a good understanding of how AI will be used in radiology. In a similar study conducted in April 2019 on three different universities in Riyadh, Saudi Arabia, approximately 50% believed they had a good understanding of AI; however, when knowledge of AI was tested using five questions, on average, only 22% of the questions were answered correctly [19]. It is shown that prior significant exposure to radiology and high confidence in knowledge and understanding of AI decrease the anxiety level towards AI [1].

Regarding ranking radiology as a potential specialty, 10.2% of participants ranked radiology without being assisted with AI as a first choice, compared to 8.6% who ranked it as a first choice when it assisted with AI. In a previous study conducted in April 2019 in Canada, out of 322 participants, 70 students considered radiology as the top specialty choice and 133 among the top three choices [1]. 13.4% ranked radiology without AI as a second choice, while 14.3% ranked it as a second choice when AI is integrated. This difference in ranking radiology with and without AI was statistically significant (P < 0.05).

About 42.9% of students who chose radiology as a first choice think that exposure to the information on AI in pre-clinical lectures will be the most beneficial way for medical students to learn about the use of AI in radiology, while 35.7% of students who chose radiology as a first choice think that information on AI in radiology rotation will be the most beneficial. This is compatible with a previous study in the United States of America in 2021, where education on AI during radiology rotations, followed by pre-clinical lectures, was the most preferred way to learn about AI [18].

When gender was considered a potential associated factor that affects specialty choice, our study demonstrated that female gender is significantly associated with a raised risk of changing radiology as a preferred future specialty due to AI by 1.9 times, this could be due to intense fear about replacement of radiologists by AI. Additionally, this might indicate that male participants are more concerned about technical challenges, society’s perception, and learning potential [20]. The recent literature suggests that the female gender is less concerned about radiology as a future specialty than men did [21-24].

In a study conducted in Brazil in February 2019, 52.5% of the surveyed students perceived AI as a threat to the radiological practice, which impacts their career choice [25]. A recent study conducted in Riyadh in 2020, revealed that half of the students believed that AI would reduce the number of radiologists needed in the future [19]. Even among radiologists who are optimistic about the future of AI-assisted radiology, there has been discussion about the possible displacement of radiologists due to productivity gains and the resultant reduction in demand for manpower due to AI [16]. One paper from the European Society of Radiology indicated that AI would not replace radiologists; in contrast, it will improve radiology and increase radiologists’ value and importance. However, radiologists have to educate themselves on AI and work with AI researchers to assure that AI is used in a way to guarantee maximum benefits to patients [26,27].

However, a survey by the American College of Radiology found a 7% decline in the demand for radiology residency programs in 2018 compared to the previous year; the impact of AI on this decrease is not yet clear based on data gathered during the Congress of the American Association of University Radiologists, held in Baltimore, April 2019 [25].

The demand for these specialists has been met in part by the rise in interest in radiology over the past few years. On the other side, medical students' sense of an AI threat to the radiology profession may unintentionally deter them from pursuing this specialization, which could have a negative demographic impact over the long term [25].

Finally, the primary findings of our study are largely in line with previously published studies conducted in Riyadh, Saudi Arabia [19], Canada [1], Germany [28], United Kingdom [2], Brazil [25], and the United States [29], which came to the conclusion that AI has a detrimental effect on student choice for radiology.

We consider this study a valuable base for evidence as it is - to our knowledge - one of the first to be conducted in the region of Saudi Arabia. Such studies can help radiology societies and radiologists to understand the extent to which AI can discourage students from considering radiology as a specialty and address the knowledge gap between students regarding AI and what could be done to fill that gap.

The study has several limitations that should be acknowledged. First, the sample of participants was drawn from a specific group of medical students at Jazan University in Saudi Arabia, which may restrict the generalizability of the findings to a broader population of medical students or different educational settings. Future studies with larger and more diverse samples are needed to improve generalizability. Second, the survey-based approach employed in the study introduces the possibility of response bias, potentially influenced by participants' personal beliefs or experiences. Moreover, the survey design and methodology may have inherent limitations that could impact the validity and reliability of the collected data. It is important to consider these potential biases when interpreting the results. Finally, the study focused on assessing AI's impact on medical students' preference for radiology as a future specialty. Still, it did not investigate certain aspects, such as the extent of AI utilization within the country or its influence on medical school curricula. Further research exploring these additional factors would provide a more comprehensive understanding of the topic.

## Conclusions

In conclusion, our analysis revealed that most students had significant exposure to AI, and the students preferred radiology as a potential specialty, but this proportion decreased when AI was integrated. We recommend that more studies on the issue be conducted to generate more evidence and data regarding this topic. Further, there is a need to organize an educational campaign; webinars and conferences to increase the awareness of medical students toward the possible implications of AI.

## Appendices

The survey used in this study:

Do you consent to participate?

o   Yes

o   No

1.     Age in years …….

2.     What is your academic year?

o   1st

o   2nd

o   3rd

o   4th

o   5th

o   6th

o   Intern

3.     Gender?

Male

Female

4.     How do you rank radiology compared to other specialties?

o   First choice

o   Second choice

o   Third choice

o   Fourth or lower choice

o   Not considering radiology

5.     If artificial intelligence did not exist, how would you rank radiology?

o   First choice

o   Second choice

o   Third choice

o   Fourth or lower choice

o   Not considering radiology

6.     Which radiology specialty are you more interested in?

o   Diagnostic

o   Interventional

o   Unsure

o   Not considering radiology 

7.     Please rate the following statements:

I have a good understanding of the field of radiology.

I have a good understanding of how artificial intelligence will be used in radiology.

I am concerned about considering radiology as a specialty due to artificial intelligence.

There will be a decrease in job opportunities for radiologists due to replacement by artificial intelligence in my lifetime.

Artificial intelligence will increase the efficiency of practicing radiologists.

8.     What exposure to radiology have you had? (Please select all that apply)

§    Pre-clinical lectures

§    Required clerkship rotation               

§    Elective rotation

§    Medical school interest group

§    Shadowing

§    Research

§    Conferences

§    None

§    Other (Please specify)

9.     Where have you heard of the use of artificial intelligence in radiology? (Please select all that apply)

§    Radiology attendings/residents

§    Non-radiology attendings/residents

§    Academic journals

§    Medical students

§    Family

§    Media/Internet

§    Research experience

§    Other

§    I have not heard of use of AI in radiology

10.  Which of the following would be the most beneficial for medical students to learn about the use of artificial intelligence in radiology with respect to their specialty choice? (Rank up to 3)
